# Supplementary material for: Self-reported symptom burden among patients attending public health care facilities in India: Looking through ICPC-3 lens
Source: PLOS Glob Public Health. 2024 May 6;4(5):e0001835. doi: 10.1371/journal.pgph.0001835 (PMC11073677; doi:10.1371/journal.pgph.0001835)
Supplement: S1 Checklist — (DOCX) [file pgph.0001835.s001.docx]

STROBE Statement—checklist of items that should be included in reports of observational studies

|  | Item No. | Recommendation | Page  No. | Relevant text from manuscript |
| --- | --- | --- | --- | --- |
| **Title and abstract** | 1 | (*a*) Indicate the study’s design with a commonly used term in the title or the abstract | 3 | This is a cross sectional study, conducted in three districts of Odisha, India |
|  |  | (*b*) Provide in the abstract an informative and balanced summary of what was done and what was found | 3 | Patients aged 18 years and above, attending the Outpatient Departments (OPD) of sampled health facilities were chosen as study participants through systematic random sampling.  Most common reasons to encounter health facilities are fever, hypertension, abdominal pain, chest pain, arthritis, skin disease, cough, diabetes, and injury. Among symptoms categories, highest patients reported for general category (29%) followed by digestive system (16% |
| Introduction | | | |  |
| Background/rationale | 2 | Explain the scientific background and rationale for the investigation being reported | 5 | If we monitor and document the symptom burden of any country, then there can be equitable planning in health care distribution by giving the area what they need.. |
| Objectives | 3 | State specific objectives, including any prespecified hypotheses | 6 | The objectives of this study were:1) to describe the socio demographics of patients presenting to public healthcare facilities; 2) to document chief complaints and reasons to visit these facilities and classify them using ICPC-3; 3) To explore difference in these complaints and ICPC-3 groups across socio-demographic and health system level |
| Methods | | | |  |
| Study design | 4 | Present key elements of study design early in the paper | 7 | This cross-sectional study was conducted in Odisha, India. |
| Setting | 5 | Describe the setting, locations, and relevant dates, including periods of recruitment, exposure, follow-up, and data collection | 7-8 | Selected districts of Odisha were Cuttack, Sambalpur and Nabarangapur with proportionate representation from each types of facilities. The only exception was Nabarangapur district as there was no medical college. Within each district, the District hospital, one SDH (if available), two CHCs and two PHCs were selected. Thus a total of three DHHs, three SDHs, six CHCs and six PHCs were covered. The data was collected for the period of three months in 2016. |
| Participants | 6 | (*a*) *Cohort study*—Give the eligibility criteria, and the sources and methods of selection of participants. Describe methods of follow-up  *Case-control study*—Give the eligibility criteria, and the sources and methods of case ascertainment and control selection. Give the rationale for the choice of cases and controls  *Cross-sectional study*—Give the eligibility criteria, and the sources and methods of selection of participants | 7 | Adult patients (more than 18 years) attending the Outpatient Departments (OPD) of sampled health facilities were chosen as study participants through systematic random sampling. |
|  |  | (*b*) *Cohort study*—For matched studies, give matching criteria and number of exposed and unexposed  *Case-control study*—For matched studies, give matching criteria and the number of controls per case | N/A |  |
| Variables | 7 | Clearly define all outcomes, exposures, predictors, potential confounders, and effect modifiers. Give diagnostic criteria, if applicable | N/A |  |
| Data sources/ measurement | 8* | For each variable of interest, give sources of data and details of methods of assessment (measurement). Describe comparability of assessment methods if there is more than one group | *8-9* | Unique chief complains were grouped and coded under ICPC-3. Patient having multiple complains were coded separately. Only chief complaints results are presented in this paper. |
| Bias | 9 | Describe any efforts to address potential sources of bias | N/A |  |
| Study size | 10 | Explain how the study size was arrived at | 7 | A total of 3377 patients was proposed to be interviewed throughout the study, with proportionate representation from each facility. It was decided to include nearly 160 patients from PHC, 240 patients from CHC, 330 patients from DHH and 850 patients from MCH, thus making grand total of 3040 from three districts. After considering non-response rate of 10%, 3377 patients was proposed to be interviewed over three months of data collection period. |

Continued on next page

| Quantitative variables | 11 | Explain how quantitative variables were handled in the analyses. If applicable, describe which groupings were chosen and why | 8 | Descriptive analysis was done to assess difference in symptom presentation across demographic indicators (age, sex, education, place of living), three levels of health care, behavioral risk factors (smoking, alcohol), and presence of chronic diseases |
| --- | --- | --- | --- | --- |
| Statistical methods | 12 | (*a*) Describe all statistical methods, including those used to control for confounding | 8 | Data visualization tools were used to explore clustering of symptoms by system. Data entry was completed in the SPSS and data cleaning, analysis was done using R software. |
|  |  | (*b*) Describe any methods used to examine subgroups and interactions | N/A |  |
|  |  | (*c*) Explain how missing data were addressed | N/A |  |
|  |  | (*d*) *Cohort study*—If applicable, explain how loss to follow-up was addressed  *Case-control study*—If applicable, explain how matching of cases and controls was addressed  *Cross-sectional study*—If applicable, describe analytical methods taking account of sampling strategy | N/A |  |
|  |  | (*e*) Describe any sensitivity analyses | N/A |  |
| Results | | | | |
| Participants | 13* | (a) Report numbers of individuals at each stage of study—eg numbers potentially eligible, examined for eligibility, confirmed eligible, included in the study, completing follow-up, and analysed | 9 |  |
|  |  | (b) Give reasons for non-participation at each stage | N/A |  |
|  |  | (c) Consider use of a flow diagram | N/A |  |
| Descriptive data | 14* | (a) Give characteristics of study participants (eg demographic, clinical, social) and information on exposures and potential confounders | 9-10 | Table 1 |
|  |  | (b) Indicate number of participants with missing data for each variable of interest | 9-10 | Table 1 |
|  |  | (c) *Cohort study*—Summarise follow-up time (eg, average and total amount) | N/A |  |
| Outcome data | 15* | *Cohort study*—Report numbers of outcome events or summary measures over time | *N/A* |  |
|  |  | *Case-control study—*Report numbers in each exposure category, or summary measures of exposure | *N/A* |  |
|  |  | *Cross-sectional study—*Report numbers of outcome events or summary measures | *11-12* | *Table 2-3 and figures* |
| Main results | 16 | (*a*) Give unadjusted estimates and, if applicable, confounder-adjusted estimates and their precision (eg, 95% confidence interval). Make clear which confounders were adjusted for and why they were included | N/A |  |
|  |  | (*b*) Report category boundaries when continuous variables were categorized | N/A |  |
|  |  | (*c*) If relevant, consider translating estimates of relative risk into absolute risk for a meaningful time period | N/A |  |

Continued on next page

| Other analyses | 17 | Report other analyses done—eg analyses of subgroups and interactions, and sensitivity analyses | N/A |  |
| --- | --- | --- | --- | --- |
| Discussion | | | | |
| Key results | 18 | Summarise key results with reference to study objectives | 15 |  |
| Limitations | 19 | Discuss limitations of the study, taking into account sources of potential bias or imprecision. Discuss both direction and magnitude of any potential bias | 17 |  |
| Interpretation | 20 | Give a cautious overall interpretation of results considering objectives, limitations, multiplicity of analyses, results from similar studies, and other relevant evidence | 18 |  |
| Generalisability | 21 | Discuss the generalisability (external validity) of the study results | 17 |  |
| Other information | |  | | |
| Funding | 22 | Give the source of funding and the role of the funders for the present study and, if applicable, for the original study on which the present article is based | 19 |  |

*Give information separately for cases and controls in case-control studies and, if applicable, for exposed and unexposed groups in cohort and cross-sectional studies.

**Note:** An Explanation and Elaboration article discusses each checklist item and gives methodological background and published examples of transparent reporting. The STROBE checklist is best used in conjunction with this article (freely available on the Web sites of PLoS Medicine at http://www.plosmedicine.org/, Annals of Internal Medicine at http://www.annals.org/, and Epidemiology at http://www.epidem.com/). Information on the STROBE Initiative is available at www.strobe-statement.org.
