# Supplementary material for: Self-reported symptom burden among patients attending public health care facilities in India: Looking through ICPC-3 lens
Source: PLOS Glob Public Health. 2024 May 6;4(5):e0001835. doi: 10.1371/journal.pgph.0001835 (PMC11073677; doi:10.1371/journal.pgph.0001835)
Supplement: S1 Questionnaire — (PDF) [file pgph.0001835.s002.pdf]

Section-I (Background Information)

1	Block Code	2	Village	3	Type of Facility:<br>[_]0 PHC [_]1 CHC [_]2 SDH<br>[_]3 DHH [_]4 MCH	4	Serial Number
5	Age in Years						
6	Sex	[_]0 Female [_]1 Male					
7	Religion	[_]0 Hindu [_]1 Islam [_]2 Christian [_]3 Others					
8	Marital Status	[_]0 Never Married [_]1 Currently Married [_]2 Separated/Divorcee<br>[_]3 Widow/Widower					
9	Ethnicity	[_]0 Schedule cast [_]1 Schedule Tribe [_]2 General					
10	Present place of living	[_]0 Urban [_]1 Semi urban [_]2 Rural					
11	Highest Education	[ ]0 Illiterate [ ]1 Primary [ ]2 High school or Secondary<br>[ ]3 Graduation and above					
12	Total expenditure per family per month(INR):						
13	Total number of family members	14. Are you the only earning member of the family? Yes/No					
15	APL/BPL (as per ration card):	[_]0 APL [_]1 BPL					
16	Current Occupation	[_] 0 Paid Work [_] 1 Self employed<br>[_] 2 Non-paid work such as volunteer [_] 3 Student<br>[_] 4 Home Maker [_] 5 Retired<br>[_] 6Unemployed (Health Reasons) [_] 7 Unemployed (Other reasons)<br>[_] 8 Others (Specify………….)					
17	Current occupation Since	……………Years					
18	Do you have any health insurance?	[_] 0 No [_]1 Yes					
19	Smoking habit [_] 0 No [_]1 Yes	Chewing tobacco [_] 0 No [_]1 Yes					
20	Height ……………. In cm<br>Weight ……………in Kg	Cholesterol …………….<br>Systolic blood pressure………………..mmhg<br>Sugar level …………………………mgdl					
21	Chief complain	Since when?					
22	Final diagnosis	Treatment given: Medical …………Surgical……………..					

Section-II Morbidity profile with severity

Arthritis	[ ]	Heart disease	[ ]	Cancer	[ ]	Osteoarthritis	[ ]
Diabetes	[ ]	Stroke	[ ]	Chronic kidney disease	[ ]	Gynecological problem	[ ]
Hypertension	[ ]	Blindness	[ ]	Epilepsy	[ ]	Hepatitis B	[ ]
Chronic lung disease	[ ]	Deafness	[ ]	Thyroid	[ ]	Injury	[ ]
Acid Peptic disease	[ ]	Dementia	[ ]	Tuberculosis	[ ]	Mental Health Disorder	[ ]
Chronic back ache	[ ]	Alcohol	[ ]	Filaria	[ ]	Skin diseases	[ ]
Dengue	[ ]	HIV/AIDS	[ ]	Malaria	[ ]	Diarrheal diseases	[ ]
Suffered diseases in last month<br>1.<br>2.<br>3.				Suffering from diseases since a year<br>1.<br>2.<br>3.			

A	When did you **last visit** a physician about this disease?<br>*Enter number of months(M) or years (Y) or Today (T)*	.....M .....Days Today<br>.....Y
B	Where did you **last visit** for this condition? [_] 1. PHC [_] 2.CHC [_] 3.SDH [_] 4.DH [_] 5.MCH [_] 6. Private	
C	Have you ever been prescribed any **medication** for this condition by a doctor?	[_] Yes [_] No [_] NA
	*If yes, continue to D. If no, go to **F***	
D	If **yes**, are you still **continuing** it?	[_] Yes [_] No [_] NA
E	If **No**, what was the reason for **stopping** it?	
F	Are you taking any medication for this condition that wasn't prescribed by a doctor or nurse (e.g. you bought it at a pharmacy or given to you by a relative)?	[_] Yes [_] No
G	Have you ever consulted **any other treatment** such as Ayuraveda/ Traditional Healer/ Faith Healer/Home Remedy etc for this condition?	
H	The number of contacts with the physician (per year) for this disease. ………………	
I	Total number of prescriptions by the physician (per year) for this disease. ………………	
J	Have you consulted any specialist? [_] Yes [_] No	
K	Total number of consultations of the specialist (per year) (of those who consulted the specialist),	
L	Were you being taken hospital admission for the disease? [_] Yes [_] No	
M	The total number of hospital admissions (per year) (of those who were admitted to the hospital),	
N	How much is this condition **limiting your activities**?<br>[_] 1. Not at all [_] 2. A little [_] 3.Somewhat [_] 4. Quite a bit [_] 5. A lot	

Expenditure for disease

Sl no	Please describe for each disease	Transport	Doctor consult	Medicine	Surgical procedure	Others	Total	Number of working days loss	Wage loss par day	Covered under insurance or not?
1										
2										
3										
4										

Section- III Quality of Well Being

This survey asks about health problems that you have experienced in the last 3 days, not including today. Please answer all questions by choosing the appropriate option

Part-I Acute and Chronic Symptoms

1. Please Indicate whether you currently experience each of the following health symptoms or problems

Do you have?		Yes	No
A	Blindness or severely impaired vision in both eyes?		
B	Blindness or severely impaired vision in only one eye?		
C	Speech problems such as stuttering or being unable to speak clearly?		
D	Missing or paralyzed hands, feet, arms or legs?		
E	Missing or paralyzed fingers or toes?		
F	Any deformity of the face, fingers, hand or arm, foot or leg, or back(e.g. severe scoliosis)		
G	General fatigue, tiredness or weakness?		
H	A problem with being under or over weight?		
I	Problems of chewing your food adequately?		
J	Any hearing loss or deafness?		
K	Any noticeable skin problems such as bad acne or large burns or scars on face, body, arms or legs?		
L	Eczema or burning/itching rash?		
	Which of the following health aides do you use/have?<br>Dentures<br>Oxygen tanks<br>Prosthesis<br>Eye glasses or contact lenses<br>Hearing aides<br>Magnifying glass<br>Neck, back or leg brace		

2. For the following list of problems Indicate which days (If any) over the past 3 days, not Including today, you had the problem. If you have not had the symptom In the past 3 days, do not leave the question blank, please fill In "no days." If you have experienced the symptom In the past 3 days, please check which of the days you had It; If you experienced It on more than one of the days, fill In all days that apply.

For example. If you had a *headache yesterday and the day before that:*

A Headache? a. No Days b. Yesterday c. 2 days ago d. 3 days ago

Do you have		No days	Y. day	2 days ago	3 days ago
A	Any problems with your vision not corrected with glasses or contact lenses (such as double vision , distorted v1sion, flashes, or floaters)?				
	Any eye pain, irritation, discharge or excessive sensitivity to lights?				
	Headache				
	Dizziness, earache, or ringing in your ears?				
	Difficulty in hearing, or discharge or bleeding from an ear?				
	Stuffy or runny nose or bleeding from the nose?				
	A sore throat, difficulty in swallowing or hoarse voice?				
	Ache or jaw pain?				
	Sore or bleeding lips, tongue, or gums?				
	Coughing or wheezing?				
	Shortness of breath or difficulty breathing?				
	Chest pain, pressure, palpitations, fast or skipped heart beat or other discomfort in the chest?				
	An upset stomach, abdominal pain, nausea, heart burn or vomiting?				
	Difficulty with bowel movements, diarrhea, constipation, rectal bleeding, black tar-like stools, or any pain or discomfort in the rectal area?				
	Pain, burning or blood in urine?				
	Loss of bladder control, frequent night-time urination, or difficulty with urination?				
	Genital pain, itching, burning! or abnormal discharge, or pelvic cramping or abnormal bleeding? (does not Include normal menstruation)				
	A broken arm, wrist, foot, leg, or any other broken bone (other than in the back)?				
	Pain, stiffness, cramps, weakness, or numbness *m the neck or back?*				
	Pain, stiffness, cramps, weakness, or numbness *m the hips or sides?*				
	Pain, stiffness, cramps, weakness, or numbness in any *of the ioints or muscles of the hand, feet, arms, or legs?*				
	Swelling of ankles, hands, feet or abdomen?				
	Fever, chills, or sweats?				
	Loss of consciousness, fainting, or seizures?				
	Difficulty with your balance, standing. or walking?				

2. The following symptoms are about your feelings, thoughts, and behaviors. Please fill In which days (It any) over the past 3 days, not Including today, you have had ...

Over the past 3 days, not Including today, you have had ...		No days	Yesterday	2 days ago	3 days ago
	Trouble falling asleep or staying asleep?				
	Spells of feeling nervous or shaky?				
	Spells of feeling upset, downhearted, or blue?				
	Excessive worry or anxiety?				
	Feelings that you had little or no control over events in your fife?				
	Feelings of being lonely or isolated?				
	Feelings of frustration, irritation, or close to losing your temper?				
	A hangover?				
	Any decrease of sexual interest or performance?				
	Confusion, difficulty understanding the written or spoken word, or significant memory loss?				
	Thoughts or images you could not get out of your mind?				
	To take any medication including over-the-counter remedies (aspirin/tylenol, allergy medications, insulin, hormones, estrogen, thyroid, prednisone)?				
	To stay on a medically prescribed diet for health reasons?				
	A loss of appetite or over eating?				

3. In the last 3 days did you have any symptoms, health complaints or pains that have not been mentioned?

Yes [_] No[_]

If yes, what were they and on which days did you have them?

Symptoms Days

Part II Self Care

Over the last 3 days (Please fill in all days that apply)		No days	Yesterday	2 days ago	3 days ago
A	Did you spend any part of the day or night as a patient in a hospital, nursing home, or rehabilitation center?				
B	Because of any impairment or health problem, did you need help with your personal care needs, such as eating, dressing, bathing or getting around your home?				

Part- III Mobility

Over the last 3 days (Please fill in all days that apply)		No days	Yesterday	2 days ago	3 days ago
A	Which days did you drive a motor vehicle?				
B	Which days did you use public transportation such as a bus, subway, Medi-van, train or airplane?				
C	Which days did you either not drive a motor vehicle or not use public transportation because of your health, or need help from another person to use?				
D	Avoid or have trouble bending over, stooping or kneeling?				
E	Have any trouble lifting or carrying everyday objects such as books, a briefcase or groceries?				
F	Have any other limitations in physical movements?				
G	Spend all or most of the day in a bed, chair, or couch because of health reasons?				
H	Spend all or most of the day in a wheelchair?				
	If in a wheelchair, on which days did someone else control its movement?				

Part- IV Physical Activity

Over the last 3 days (Please fill in all days that apply)		No days	Yesterday	2 days ago	3 days ago
A	Have trouble climbing stairs or inclines or walking off the curb?				
B	Avoid walking, have trouble walking, or walk more slowly than other people your age?				
C	Limp or use a cane, crutches or walker?				
D	Avoid or have trouble bending over, stooping or kneeling?				
E	Have any trouble lifting or carrying everyday objects such as books, a briefcase or groceries?				
F	Have any other limitations in physical movements?				
G	Spend all or most of the day in a bed, chair, or couch because of health reasons?				
H	Spend all or most of the day in a wheelchair?				
	If in a wheelchair, on which days did someone else control its movement?				

Part-V Usual Activity

Over the last 3 days (Please fill in all days that apply)		No days	Yesterday	2 days ago	3 days ago
A	Because of any physical or emotional health reasons, on which days did you avoid, need help with or were limited in doing some of your usual activities, such as work, school or housekeeping?				
B	Because of any physical or emotional health reasons on which days did you avoid or feel limited in doing some of your usual activities, such as visiting family or friends, troubles, shopping, recreational or religious activities?				
C	On which days did you have to change any of your palns or activities because of your health? (Consider A question only?)<br><br>If limited, please describe:				

Would you say that your health is?

Excellent		Very Good		Good		Fair		Poor	

Compared to a year ago, how would you rate your health in general now?

Much better than a year ago	
Somewhat better than one year ago	
About the same as a year ago	
Somewhat worse than a year ago	
Much worse than a year ago	

Think about a scale of 0 to 100, with zero being the least desirable state of health that you could imagine and 100 being perfect health. What number, from 0 to 100 would you give to the state of your health. On average, over the last 3 days?

0	10 20 30 40 50 60 70 80 90 100
