# Supplementary material for: Self-reported symptom burden among patients attending public health care facilities in India: Looking through ICPC-3 lens
Source: PLOS Glob Public Health. 2024 May 6;4(5):e0001835. doi: 10.1371/journal.pgph.0001835 (PMC11073677; doi:10.1371/journal.pgph.0001835)
Supplement: Attachment — Submitted filename: PGPH-D-23-00452-HTN.docx [file pgph.0001835.s004.docx]

Dear Dr Sanghamitra,

Before going into detail on reading the content of your submission, I would like to point out a few things:

- What is missing is the argumentation for why you want to use the ICPC-3 for your study? It is mentioned in the introduction, but it bares no evidence. Why use ICPC-3 and not ICPC-2 or ICD01 or ICD-11 or SNOMED CT? Please make your choice more clear.

- There is the mention in the introduction for the WICC being the developers of the ICPC-3: this is not the case. The ICPC-3 has been developed by the ICPC-3 Consortium, not by WICC. You can read about that on the [ICPC-3.info](https://ICPC-3.info) website.

- What is missing is the most basic reference" the ICPC-3 Manual and Classification, which is the book.

- The conclusion is not reflecting the results of the study in terms of answering the research questions.

I think your research question could be: Is the ICPC-3 suited for exploring and comparing across and between different levels of health care? The study should be answering this question.
