# Supplementary material for: Self-reported symptom burden among patients attending public health care facilities in India: Looking through ICPC-3 lens
Source: PLOS Glob Public Health. 2024 May 6;4(5):e0001835. doi: 10.1371/journal.pgph.0001835 (PMC11073677; doi:10.1371/journal.pgph.0001835)
Supplement: Attachment — Submitted filename: Reviewers comments and responses.docx [file pgph.0001835.s005.docx]

**Response to reviewers’ comments on** PGPH-D-23-00452: “Self-reported symptom burden among patients attending public health care facilities in India: Looking through ICPC-3 lens”

We thank the editor and reviewers for their constructive comments which we feel have helped us strengthen this manuscript. Given below are the point-by-point responses in italics to the comments. Accompanying this document, we are attaching two copies of the manuscript, one with tracked changes and another a clean version. We look forward to hearing a positive response.

Reviewer 1

What is missing is the argumentation for why you want to use the ICPC-3 for your study? It is mentioned in the introduction, but it bares no evidence. Why use ICPC-3 and not ICPC-2 or ICD01 or ICD-11 or SNOMED CT? Please make your choice more clear.

*Response: Addressed this by adding justification in the introduction to use ICPC-3, having advantages over other classification systems.*

*We added following line in page 6 and 7 of manuscript*

*“The latest version of the International Classification of Primary Care (ICPC-3), published in 2021. (10) represents a substantial improvement over the previous edition (11). Unlike its predecessors, ICPC-3 caters to all primary care clinicians, not just family physicians or general practitioners. Its interoperability with various classifications, such as ICD-10, ICD-11, ICHI, ICF, and SNOMED CT, allows seamless integration and information exchange. Embracing ICPC-3 offers a logical and comprehensive approach to organizing and conceptualizing primary care within a unified classification system. (12)This study examines the suitability of ICPC-3 for cross-level exploration and comparison of healthcare services.”*

There is the mention in the introduction for the WICC being the developers of the

ICPC-3: this is not the case. The ICPC-3 has been developed by the ICPC-3

Consortium, not by WICC. You can read about that on the ICPC-3.info website.

*Response. We have edited the statement by changing WICC with ICPC-3 Consortium as the developer of the ICPC-3 on page 6 of manuscript*.

What is missing is the most basic reference & quot; the ICPC-3 Manual and Classification, which is the book.

*Response: We have added the manual book as reference in the introduction as reference 10*

The conclusion is not reflecting the results of the study in terms of answering the

research questions.

*Response: We have added following line in the conclusion*

*“This is the first Indian study using ICPC-3 classification for all the three levels of health care. Irrespective of age, socio economic variables and tiers of healthcare top three groups to visit public health facility according to ICPC-3 classification were consistent i.e., general, digestive, and circulatory*”

I think your research question could be: Is the ICPC-3 suited for exploring and

comparing across and between different levels of health care? The study should be

answering this question.

*Response: As per suggestion, we have added the statement “This study examines the suitability of ICPC-3 for cross-level exploration and comparison of healthcare services.” in the introduction*

Reviewer #2: This is one of the first attempt to use International Classification of Primary Care in health care facilities of India. The author need to discuss following for better understanding

a. How sample size was estimated and how proportions were estimated from each facility (Line no. 145- 150)

*Response: This study was part of government project and sample size calculation was done for that project, but as this data was collected first time we used this opportunity to generate some data in this nascent field. We have added following on page 8 in discussion as study limitation*

*“As the study is a part of one of the government project, the sample size calculation is not based on ideal statistical based sample size calculation but based on the project objectives”*

b. how coding was done and aspects of inter- rater reliability must be discussed

*Response: The coding was done by two experienced researchers and validated with the senior researcher for any discrepancies. We have added this line in method section on page 8 of manuscript*.

c. Does ICP classification provides any additional advantage over ICD classification in Indian setting

*Response: In Indian system in primary healthcare facilities primarily addresses clinical symptoms rather than diagnosis, that’s why ICP have advantage over ICD*

d. As fever is one of predominant symptoms , what seasons / months were chosen for data collection from health facility ? Does this choice of month may impact data collection in form of symptoms especially ?-

*Response: We agree to the reviewer’s comment that the month of data collection can affect fever symptoms and its prevalence, but as this study was part of a larger government project we didn’t had the flexibility to choose different season and months this is a limitation and we have added it in discussion*.

e. Line no. 177-patients with multiple complaints ... separately ".. where this data is provided ?
*Response: We have removed the sentence as only patient with chief complaints were analysed*

f. stratification of diseases and symptoms in Table 2 should be done as per health facility rather as separate column

*Response: We have constructed this table to compare three facility levels as this was the third objective of the study*

g. How data collection was done from SDH / DH / Medical college - as they have multiple OPDs .. so which area was targeted for one week data collection by investigators ?

*Response: We have done data collection from general medicine department OPDs*
