# Supplementary material for: Self-reported symptom burden among patients attending public health care facilities in India: Looking through ICPC-3 lens
Source: PLOS Glob Public Health. 2024 May 6;4(5):e0001835. doi: 10.1371/journal.pgph.0001835 (PMC11073677; doi:10.1371/journal.pgph.0001835)
Supplement: Attachment — Submitted filename: Reviewers comment.docx [file pgph.0001835.s007.docx]

**Response to reviewers’ comments on** PGPH-D-23-00452: “Self-reported symptom burden among patients attending public health care facilities in India: Looking through ICPC-3 lens”

We thank the editor and reviewers for their constructive comments which we feel have helped us strengthen this manuscript. Given below are the point-by-point responses in italics to the comments. Accompanying this document, we are attaching two copies of the manuscript, one with tracked changes and another a clean version. We look forward to hearing a positive response.

Reviewer’s 1 I recommend having the submission reviewed on the use of proper English and punctuation which will make it more readable. Some lines have been mentioned below.

It is clearly reflecting the complexity of the Health Care System in India and how ICPC-3 is supporting the purpose it has been designed for: the importance of primary level of care and the exchange of data between tiers from a Primary Health Care perspective.

However, this is not yet reflected in the study objectives in the introduction and is also missing in the conclusion. It pops up somewhere in line 133.

Thank you for your suggestion we have revised the line 133.

Line 46, 47: add more keywords referring to the content of the article

Added key words: symptoms burden in Indian health system

Line 98: remain

Line 99: has

Line 102: primary care, not the primary care

Line 105: need or needs?

Please check the English in this article on correctness

Line 110: it is not updated but reviewed and launched in December 2020

Line 117, 118: The sentence starting with It not only saves …..what does it mean?

Line 133: The study examining the suitability of ICPC-3 for cross-level exploration is not mentioned in the study objectives in the introduction. It should also be part of the conclusion, stating if the ICPC-3 has shown to be suitable, and why.

Line 155: was proposed = were proposed or were interviewed…again check correctness of English

Line 165: fidelity is not the correct word, should be validity?

Line 170: had already

Line 185,186: not very understandable sentence

Line 222 and 234 : reasons for encounter

Line 224: have not been reported?

Again please check for English

Line 237, 238, 239: Within sex both male and female present highest in

circulatory system whereas females in addition have pregnancy and childbearing related

visits which in male is musculatory reasons in the second position.

Should this read as:

Within sex, both male and female present highest in

circulatory system whereas females in addition have pregnancy and childbearing related

visits, and for male is musculatory reasons in the second position?

A lot of punctuation is missing in all of the article.

Line 275: majority of

Line 281 and 282: Punctuation

Line 303: But not to start a sentence.

Line 321: Starting a sentence with So…….maybe use: We recommend ….

Line 328: has shown or studies have shown

Line 338: reasons for encounter

Thank you for suggestion, we have improved the punctuation and overall English in whole article.
