# Supplementary material for: Self-reported symptom burden among patients attending public health care facilities in India: Looking through ICPC-3 lens
Source: PLOS Glob Public Health. 2024 May 6;4(5):e0001835. doi: 10.1371/journal.pgph.0001835 (PMC11073677; doi:10.1371/journal.pgph.0001835)
Supplement: Attachment — Submitted filename: Reviewers comments.docx [file pgph.0001835.s008.docx]

**Response to reviewers’ comments on** PGPH-D-23-00452: “Self-reported symptom burden among patients attending public health care facilities in India: Looking through ICPC-3 lens”

We would like to express our gratitude for the opportunity to revise and resubmit our manuscript titled "Self-reported symptom burden among patients attending public health care facilities in India: Looking through ICPC-3 lens" in response to the feedback provided by the reviewers. We sincerely appreciate the time and effort invested by the reviewers and the editorial team in evaluating our work.

We are pleased to report that since our initial submission, our manuscript has undergone significant revisions based on the constructive feedback received from the initial peer review process. We carefully addressed all concerns raised by the first two reviewers and made substantial improvements to the manuscript accordingly. Specifically, we focused on enhancing clarity, providing additional contextualization, and strengthening the argumentation of our findings.

In our correspondence dated 27th October 2023, we were informed that the only outstanding requirement for publication was the inclusion of a data availability statement, which we promptly provided. However, we were surprised to receive additional reviewer comments, particularly from a third reviewer, in the most recent communication.

While we acknowledge the importance of addressing all reviewer comments, we must express our reservations regarding the feasibility of accommodating the suggestions put forth by the third reviewer. The concerns raised by this reviewer require significant changes to the methodology, results, and discussion sections of the manuscript. Upon careful consideration, we believe that implementing these suggestions would not only necessitate extensive revisions but also fundamentally alter the structure and focus of the paper.

Given that the manuscript has already undergone a prolonged review process spanning more than 1.5 years, we kindly request your perspective on this matter. We are fully committed to addressing any minor changes suggested by the third reviewer that align with the scope and objectives of our study. However, we believe that accommodating the extensive revisions proposed by the third reviewer may not be feasible within a reasonable timeframe and could compromise the integrity of the manuscript.

We trust that you will consider our perspective and provide guidance on how best to proceed with the review process. We remain open to further discussion and are committed to working collaboratively to ensure the successful publication of our research.

Thank you for your attention to this matter.

Reviewer #1: Although the manuscript has improved a lot, the abstract still needs a slight improvement of English.
As for the use of "reason for visits": this needs to be Reason for Encounter to be in line with the principles of the ICPC-3. I think an overactive translator has changed reason for encounter into reason for visits. Please correct this.

Corrected in abstract

What is still missing is the experience of using ICPC-3 for the research. Is ICPC-3 an improvement for registrations or not? That needs to be made clear. Although it is not the research question it can be mentioned in the conclusion that the ICPC-3 is indicated for this kind of research.

We have added this in the conclusion

I also still miss the questionnaire that has been used. Without it it is hard to see what the results are based on.

Questionnaire attached for your reference.

Minors/format issues:
1. Line 52, 55, etc: In the abstract, the full name for abbreviations should be shown when appearing for the first time

Corrected

2. Line 148, 340: Unexplained abbreviation (NCD, ADL)

Corrected

3. Line 343: Duplicated "Secondly"

Corrected

4. There are many typos and formatting mistakes, please carefully check the manuscript.

Corrected
